# The impact of infectious disease experience on household consumption: evidence from rural China

**DOI:** 10.3389/fpubh.2024.1390432

**Published:** 2024-06-12

**Authors:** Linlin Han, Xiaoling Xue, Jinxiang Yu

**Affiliations:** ^1^School of Economics, Shandong Normal University, Jinan, Shandong, China; ^2^School of Economics and Management, Qilu Normal University, Jinan, Shandong, China; ^3^School of Business, University of Jinan, Jinan, Shandong, China; ^4^School of Economics and Management, Nanjing Agricultural University, Nanjing, Jiangsu, China

**Keywords:** infectious disease experience, household consumption, rural residents, risk response, instrumental variable (IV) method

## Abstract

**Objective:**

The issue of low consumption among rural households in China has a longstanding history, and the experience of infectious diseases may exacerbate the existing challenges in fostering consumption growth. However, studies that characterize the impact of infectious diseases on household consumption are limited in China. This study aims to explore rural household consumption responses to infectious diseases post-assessment, and identify the underlying mechanisms.

**Methods:**

A total of 1,539 rural households from China Family Panel Studies (CFPS) datasets of 2014, 2016, 2018, and 2020 were recruited as the study sample. The presence of infectious disease experience was employed as the independent variable and household consumption as the dependent variable. A panel fixed effects (FE) regression model was initially employed to identify the influence of infectious disease experiences on rural household consumption. The instrumental variable (IV) method was used to address potential endogeneity between independent and dependent variables. Robustness checks such as Propensity Score Matching (PSM) test were employed to ensure the reliability of the findings.

**Results:**

The results reveal a statistically significant negative impact of infectious disease experiences on consumption over time, becoming no more significant at around 7–9 years post-disaster. This effect leads to more pronounced consumption deprivation for households with limited health insurance coverage and heightened healthcare resource constraints. The mechanism test indicates that infectious disease experiences affect the consumption levels of rural households through channels that include income constraints, the crowding-out of healthcare expenditure, and risk perception, with the precautionary savings motive acting as a moderator. Furthermore, the diminishing effect of infectious diseases on individual consumption surpasses that of natural disasters. Temporal discrepancy is observed in the impacts of infectious and chronic disease shocks on household consumption. The accumulation of liquid assets emerges as an effective strategy for households to mitigate the impact of infectious disease shocks.

**Conclusion:**

The findings underscore the importance of integrating short- and long-term policies to bolster consumption capacity, strategically allocate inter-regional medical resources, and fortify the resilience of rural households against economic risks.

## 1 Introduction

Consumption stands as a pivotal endogenous factor, integral to enhancing life quality and propelling macroeconomic advancement. Despite its significance, in China, household consumption demand has been lagging, with total savings as a percentage of GDP surpassing not only those of developed economies like the UK and the US but also those of developing nations such as India and Vietnam, as per World Bank figures (1). The 14th Five-Year Plan underscores the imperative to invigorate domestic demand to meet the populace's aspirations for a superior quality of life. However, the recent pandemic has further constrained effective consumption demand.

Rural households, marked by modest wealth and precarious social safety nets, confront greater challenges in managing risks associated with income volatility, unemployment, health emergencies, and unforeseen expenditures (2). This vulnerability exacerbates the low consumption predicament in rural regions, making it imperative to bolster consumer demand to elevate the general welfare of rural households.

Infectious diseases, once regarded as rare events with minimal repercussions, have seen a significant rise in both frequency and impact in recent years. This trend poses a substantial challenge to modern societies due to the inherent nature of communicable diseases. Initially, these diseases are transmissible, spreading rapidly across geographical boundaries. According to data from the World Health Organization, the rapid spread of COVID-19 has affected over 200 countries and territories, with more than 760 million confirmed cases worldwide (3). Secondly, they erode economic value, causing not only a decrease in immediate income but also impairing long-term labor productivity, resulting in financial risk to the household (4–8). Lastly, they are prone to information uncertainty and can incite panic (9–11). The outbreak of infectious diseases triggers widespread anxiety and fear, which are exacerbated by reduced expected income and an increased tendency to save (12). This, in turn, significantly influences consumer expectations and confidence. Consequently, exploring the effects of infectious diseases on rural household consumption and their underlying mechanisms carries substantial policy implications for unlocking the potential of household consumption and fostering integrated urban and rural economic growth.

Despite an increasing body of research on the impact of infectious disease experiences on household consumption, the conclusions remain inconsistent. One viewpoint posited that the negative exogenous shock of infectious diseases impairs the health of those affected, leading to a decline in household labor income and an increase in spending on healthcare and disease prevention measures (5). This situation could engender a greater sense of risk, inciting panic and herd mentality, which in turn may lead to an increased propensity for precautionary saving and a subsequent reduction in current household consumption (13–15). On the other hand, an alternative perspective posited that exposure to infectious diseases may heighten individuals' awareness of life's fragility, activating defense mechanisms on both psychological and behavioral levels (16, 17). This was evidenced by a focus on present utility, a short-term rise in the demand for material goods and wealth, a tendency toward risk-taking, and a preference for premium products (18). These behavioral changes, driven by a desire to seize the moment and secure immediate satisfaction, could potentially lead to an overall increase in current consumption. Moreover, there is a lack of research specifically exploring the short-term and persistent causal effects of infectious disease experience on household consumption from a micro-household viewpoint.

Our paper posits three hypotheses regarding the impact of infectious diseases on household consumption. Hypothesis 1 suggests that infectious diseases, due to their inherent risk and potential for impairment, lead to a decrease in consumption levels among rural households. The immediate negative income shock and increased risk aversion resulting from these diseases compel households to cut back on spending and increase savings, thereby reducing short-term consumption (19–21). Hypothesis 2 explores the temporal dynamics of this impact, proposing that the effects of infectious disease shocks on household consumption are likely to diminish over time. The rationale is that while the initial shock may cause a significant drop in consumption, over time, factors such as recovery from the disease, adjustments in consumption habits, and economic resilience-building measures can lead to a gradual normalization of consumption patterns (22, 23). Lastly, Hypothesis 3 delves into the mechanisms through which infectious diseases influence household consumption. It identifies four key channels: the income constraint effect, where labor disruptions and health declines reduce income and tighten budget constraints; the medical expenditure crowding-out effect, where increased healthcare costs lead to reduced spending on non-medical goods and services; the risk perception effect, which causes anxiety and prompts a reduction in discretionary spending; and the precautionary savings effect, where heightened risk aversion leads to increased savings at the expense of current consumption. These mechanisms collectively contribute to a significant reduction in the consumption levels of rural households following an outbreak of infectious diseases.

This study aims to explore rural household consumption responses to infectious diseases post-assessment and identify the underlying mechanisms through a more nuanced and comprehensive micro-perspective. It utilizes data from the 2014, 2016, 2018, and 2020 China Family Panel Studies (CFPS) for empirical analysis. To mitigate issues of endogeneity and self-selection, the study applies instrumental variables and additional methodological approaches. The results reveal a notable deterrent effect on consumption as a result of exposure to infectious diseases, with this effect diminishing over time. The negative impact of infectious disease shocks on consumption is more pronounced for households with limited health insurance coverage and those facing greater constraints in accessing healthcare resources. The mechanisms through which infectious diseases impact household consumption encompass income constraints, crowding out of healthcare expenditures, and risk perception, with the precautionary savings motive playing a moderating role. The impact of infectious diseases on household consumption differs significantly from that of frequent natural disasters, such as droughts and floods, and chronic disease shocks. Furthermore, the study underscores the heterogeneous risk aversion effects in households' use of risk buffers in response to infectious disease shocks.

This paper makes a valuable contribution to the field by conducting an empirical analysis that examines the impact of experiences with infectious diseases on rural household consumption. It delves into the varied effects of healthcare resource utilization on these impacts, providing policy-relevant insights aimed at enhancing consumer welfare and optimizing healthcare resource allocation, particularly in economically disadvantaged and remote regions during the post-epidemic period. Furthermore, the research evaluates the heterogeneous effects of different time frames, disaster types, and disease categories on household consumption patterns. It also assesses the efficacy of various risk-buffering strategies in mitigating the effects of infectious disease shocks, thereby enriching our understanding of the dynamics of household consumption following such events.

## 2 Materials and methods

### 2.1 Data processing

This study leveraged the China Family Panel Studies (CFPS) database from 2014, 2016, 2018 and 2020 as the research sample. Conducted by the China Social Science Survey Center of Peking University, the CFPS project extensively documented household finances, consumption status, and demographic characteristics at three levels: individual, family, and community (24). Importantly, the 2014 CFPS questionnaire included information on the occurrence of infectious diseases and natural disasters in rural areas. We concentrated on households that participated in the 2014 survey and tracked their relevant information until 2020. Data processing involved designating the “financial respondent” as the head of the household agent and aligning it with family and community information. Since the CFPS questionnaire specifically addressed infectious diseases and natural disasters in rural areas, this paper retained only the rural sample. To construct a four-period balanced panel dataset, data were combined and matched based on the household ID. Furthermore, Exclusion criteria were applied, removing samples with household consumption, income, and asset amounts less than or equal to zero, as well as samples with missing dependent, independent, or control variables, or marked as “don't know” or “refused to answer.” For consistency, this study retained data only for household heads aged between 16 and 80. As a result, the final dataset comprised 6,156 sample entries, covering 1,539 rural households across the four survey phases.

### 2.2 Construction of indicators

#### 2.2.1 Dependent variable

The dependent variable in this study is household consumption. Drawing on previous studies, Household Consumption 1 encompasses total annual expenditure on household consumption, including spending on food, clothing, housing, travel, household appliances and services, communication, culture and recreation, healthcare, and education (25). Household Consumption 2 is defined as annual household consumption expenditure excluding education and healthcare consumption. The rationale behind this exclusion is twofold: the unpredictable nature of healthcare costs and the significant, predetermined expenditure on education for households with school-aged children. The empirical data on household consumption undergo logarithmic transformation. To enhance the robustness of the analysis, a robustness test is conducted using Household Consumption 2 as a proxy for Household Consumption 1.

#### 2.2.2 Independent variable

Following Li and Li (10), the independent variable in this study is the presence or absence of infectious disease experience within a household. A dummy variable is introduced, which takes a value of 1 if the household has encountered an infectious disease and 0 otherwise. It is important to highlight that the detailed investigation into whether the villages where the households were situated had experienced infectious diseases or natural disasters, such as droughts, floods, and forest fires, from 2010 to 2013, was exclusively conducted in the CFPS 2014 questionnaire.^1^ Therefore, this paper assesses the short-term and persistent impact of infectious disease experience on rural households' consumption levels.

#### 2.2.3 Control variable

This study employs a set of control variables, encompassing household head characteristics, family attributes, and regional factors. The set of household head characteristics includes gender, age, ethnicity, marital status, education level, health status, frequency of weekly exercise, time preference, and risk preference. To account for the potential non-linear impact of age on household consumption, the study includes the variable age squared/100 (26). Family attributes comprise variables such as family size, number of children under 15 years old, number of older adult over 60 years old, annual household income, property value, number of owned properties, value of financial assets, participation in pension and health insurance programs, and self-assessed social status. Logarithmic transformations are applied to household income and the value of each asset type. Economic and social status may influence consumption decisions through subjective family sentiments, necessitating control to mitigate omitted variable bias (27). Additionally, the paper controls for the logarithm of per capita GDP of the province to which the household belongs and includes regional variable for the eastern, central, and western regions based on the household's geographical location. Descriptive statistics for each variable are presented in Table 1.

**Table 1:** Descriptive statistics of variables.

**Variable**	**Definition**	**Mean**	**SD**
Consum1	Total annual consumption expenditure of the log household	9.900	0.541
Consum2	Log annual consumption expenditure excluding education and health expenditure	9.716	0.530
Epidemic	1 for experienced an infectious disease, 0 for others	0.035	0.053
Edu	The education level of the head is assigned a value of 0–7 from low to high	1.009	1.103
Gender	1 for males and 0 for females	0.50	0.500
Age	Age of head of household	47.65	15.48
Age2	Age of head of household squared/100	25.08	14.92
Ethnic	1 for Han Chinese, 0 for ethnic minorities	0.97	0.158
Marital	1 for married, 0 for others	0.83	0.378
Health	The subjective health evaluation of the head is assigned a value of 1–5	2.99	1.24
Exercise	Frequency of weekly exercise	1.698	2.20
Timepre	Willingness to live in the present	2.96	1.61
Riskpre	Willingness to take investment risks	3.08	0.95
Number	Number of permanent residents in the household	3.55	1.50
Kid	Number of people under 15 years old in the household	0.74	0.67
Older	Number of people in households over 60 years old	0.74	0.68
Income	Log of total annual household income	10.225	0.538
Property	Log of total value of household-owned properties	13.087	0.612
Pnumber	Total number of owned properties in the household	1.216	0.518
Asset	Log of total value of household-owned finance asset	8.997	0.527
Pension	1 for participating in old-age insurance, 0 for others	0.522	0.428
Medical	1 for participating in medical insurance, 0 for others	0.934	0.248
Status	Self-assessed economic and social status of the household	3.088	1.045
perGDP	Gross regional product per capita in logarithmic provinces	1.758	0.929
Area	Whether the household is located in the eastern, central, or western part of China	1.843	0.833

### 2.3 Empirical models

This study utilizes balanced panel data to examine the association between infectious disease experiences and household consumption. To address potential bias from omitted variables, it is crucial to control for unobserved heterogeneity that varies across households and over time. Furthermore, given the correlation between these unobserved characteristics and independent variables like infectious diseases, a fixed effects model is selected for the analysis (26). To ensure methodological rigor, a Hausman test was performed on the baseline regression model (*F* = 48.91, *p*-value = 0.00), corroborating the suitability of the fixed effects method. The regression model is detailed as follows:


(1) $$\begin{array}{c} consum_{ijt}\;=\;a_{0}\;+\;a_{1}epidemic_{ij}\;+\;a_{2}X_{ijt}\;+\;a_{3}Y_{ijt}\;+\;a_{4}Z_{jt}\;+\\ \lambda_{j}\;+\;\eta_{t}\;+\;u_{ijt} \end{array}$$

In Equation (1), *consum*_*ijt*_ denotes the natural logarithm of household consumption for the *ith* household in county *j* during period *t*. The *epidemic*_*ij*_ is a dummy variable indicating whether the *ith* household's village in county *j* has experienced an infectious disease. *X*_*ijt*_, *Y*_*ijt*_, and *Z*_*jt*_ represent the head-of-household, household, and district control variables, respectively. λ_*j*_ represents household fixed effects, which account for unobservable, time-invariant household-specific heterogeneities that could influence consumption. η_*t*_ denotes year fixed effects, capturing time-variant factors that may uniformly affect all households across the study period. *u*_*ijt*_ is a random error term, and robust standard errors are clustered at the household level.

To investigate the impact mechanisms of infectious disease experiences on household consumption, this study introduces additional dependent variables such as income, medical expenditure, transportation, tourism, and entertainment consumption expenditures. It aims to examine the income constraint effect, medical expenditure crowding-out effect, and the risk perception effect, and tests the mechanism of the precautionary savings effect using a moderating effect model. The empirical models are constructed as follows:


(2) $$\begin{array}{c} inc_{ijt}\;=\;b_{0}\;+\;b_{1}epidemic_{ij}\;+\;b_{2}X_{ijt}\;+\;b_{3}Y_{ijt}\;+\;b_{4}Z_{jt}\;+\\ \lambda_{j}\;+\;\eta_{t}\;+\;{u_{ijt}}^{1} \end{array}$$

(3) $$\begin{array}{c} exp_{ijt}=c_{0}+c_{1}epidemic_{ij}+c_{2}X_{ijt}+c_{3}Y_{ijt}+c_{4}Z_{jt}+\\ \lambda_{j}+\eta_{t}+{u_{ijt}}^{2} \end{array}$$

(4) $$\begin{array}{c} ris_{ijt}=d_{0}+d_{1}epidemic_{ij}+d_{2}X_{ijt}+d_{3}Y_{ijt}+\\ d_{4}Z_{jt}+\lambda_{j}+\eta_{t}+{u_{ijt}}^{3} \end{array}$$

(5) $$\begin{array}{c} consum_{ijt}=e_{0}+e_{1}epidemic_{ij}+e_{2}epidemic_{ij}\times pre_{ijt}+e_{3}inc_{ijt}+\\ e_{4}exp_{ijt}+e_{5}ris_{ijt}+e_{6}X_{ijt}+e_{7}Y_{ijt}+\\ e_{8}Z_{jt}+\lambda_{j}+\eta_{t}+{u_{ijt}}^{4} \end{array}$$

In Equation (2), *inc*_*ijt*_ represents the logarithm of the annual total income of the household, while *exp*_*ijt*_ denotes the logarithm of the annual medical expenditure of the household in Equation (3). The risk perception variable, *ris*_*ijt*_ is utilized as a proxy for risk perception factors influenced by infectious diseases, leading household to spontaneously reduce activities such as dining out, and cultural and recreational activities to minimize the probability of exposure to public places (9). Hence, transportation, tourism, and entertainment expenditures are employed as negative proxy variables for risk perception. According to whether the head is employed and the source of income, the sample is divided into an agricultural production group, a wage-earning group, and a self-employment group. Referring to Yang et al. (25), the variable *pre*_*ijt*_ takes values 1–3 based on the precautionary savings motivation faced by the different household groups, with the scale decreasing from larger to smaller. Equation (2) explores the income-constraining effect with *b*_1_ < 0 indicating that infectious diseases reduce household income. Similarly, *c1* > *0* in Equation (3) measures the healthcare expenditure crowding-out effect of infectious diseases, and *d1*<*0* in Equation (4) measures the risk perception effect of infectious diseases. Finally, the moderating role of precautionary savings in the experience of infectious diseases is examined by testing the sign and significance of the *e*_2_ coefficient in Equation (5).

## 3 Results

### 3.1 Benchmark regression results

Table 2 presents the results of the baseline regression examining the impact of infectious disease experience on household consumption. In Column (1), the estimated coefficient is significantly negative at the 1% significance level. Columns (2) and (3) progressively incorporate control variables for the head, family, regional characteristics, and fixed effects. The estimated coefficients remain significantly negative at the 1% significance level, indicating a short-term reduction in consumption due to infectious diseases. The stability of the coefficients after the inclusion of controls indicates the exogeneity of the disease impact on consumption. Columns (4) to (6) depict regression results by replacing household consumption 1 with household consumption 2 as the dependent variable. The observed effects of infectious diseases on household consumption levels are consistently significantly negative at the 1% significance level, affirming the presence of a suppressive effect of infectious disease experience on household consumption. According to the baseline regression results in columns (3) and (6), infectious disease experience significantly reduces overall household consumption expenditure in the 1–3 years post-disaster by 6.3% at the 5% significance level. Moreover, when excluding medical and educational costs, household consumption declines by 6.1% at the 1% significance level, thereby validating Hypothesis 1.

**Table 2:** Benchmark regression results.

	**(1)**	**(2)**	**(3)**	**(4)**	**(5)**	**(6)**
	**consum1**			**consum2**		
Epidemic	−0.223^***^	−0.070^***^	−0.063^***^	−0.102^***^	−0.076^***^	−0.061^***^
	(−3.84)	(−2.90)	(−3.55)	(−4.87)	(−3.60)	(−3.34)
Gender		−0.040^***^	−0.052^***^		−0.039^***^	−0.047^***^
		(−2.34)	(−3.12)		(−2.45)	(−2.86)
Age		0.008	0.009^*^		0.009^*^	0.007
		(1.56)	(1.90)		(1.71)	(1.42)
Age2		−0.008	−0.022^***^		−0.010	−0.011^***^
		(−1.56)	(−4.68)		(−1.20)	(−3.32)
Ethnic		−0.318^***^	−0.294^**^		−0.344^***^	−0.295^**^
		(−2.97)	(−2.45)		(−3.14)	(−2.76)
Marital		0.241^***^	0.468^***^		0.244^***^	0.234^***^
		(9.34)	(10.80)		(10.08)	(9.88)
Edu		0.053^***^	0.065^***^		0.063^***^	0.064^***^
		(6.89)	(7.92)		(7.88)	(7.74)
Health		−0.024^***^	−0.021^***^		−0.008	−0.022^***^
		(−3.60)	(−3.37)		(−1.28)	(−3.12)
Exercise		0.027^***^	0.030^***^		0.027^***^	0.030^***^
		(6.92)	(7.08)		(6.96)	(7.12)
Timepre		0.033^***^	0.005		0.004	0.005
		(4.68)	(0.68)		(0.54)	(0.65)
Riskpre		0.015	0.013		0.014	0.001
		(0.88)	(1.32)		(1.42)	(0.18)
Number			0.184^***^			0.182^***^
			(10.89)			(10.67)
Kid			0.034^**^			0.034^**^
			(2.45)			(2.49)
Older			0.050^***^			0.052^***^
			(3.92)			(4.13)
Income			0.110^***^			0.099^***^
			(7.44)			(6.64)
Property			0.043^**^			0.039^**^
			(2.37)			(2.28)
Pnumber			0.089^***^			0.092^***^
			(7.79)			(7.98)
Asset			0.016			0.001
			(0.32)			(0.02)
Pension			0.062^***^			0.063^***^
			(7.39)			(7.20)
Medical			0.026^***^			0.035^**^
			(2.87)			(2.16)
Status			0.019^***^			0.016^***^
			(3.55)			(4.41)
perGDP			0.021^***^			0.023^***^
			(2.78)			(2.66)
Area			−0.018^**^			−0.017^**^
			(−2.29)			(−2.03)
FE	No	No	Yes	No	No	Yes
Obs	1,539	1,539	1,539	1,539	1,539	1,539
*R*-squared	0.189	0.213	0.219	0.174	0.250	0.251

^***^, ^**^, ^*^ indicate significance levels of 1%, 5%, and 10%, respectively; *t*-values for standard errors clustered to the household level are in parentheses.

Concerning the control variables, the quadratic age term exhibits a negative impact on household consumption, indicating an inverted U-shaped relationship between age and household consumption levels, with middle-aged households typically having higher consumption. Holding other factors constant, households headed by females, with married marital status, belonging to ethnic minorities, and possessing higher levels of education and health status, demonstrate higher consumption levels. An increase in household size or the number of children and older adult in the household leads to a significant rise in consumption expenditure, reflecting the higher costs of childrearing and older adult care. Both the value and the quantity of properties exert a positive influence on household consumption, reflecting a wealth effect. Rural households participating in pension or medical insurance schemes exhibit a greater inclination toward consumption compared to their non-participating counterparts. Additionally, consumption expenditures witness a significant increase with the elevation of the household's social status, possibly attributed to social expenditures (27).

### 3.2 Robustness tests

To test the robustness of the empirical results, the study conducts a series of robustness checks.

#### 3.2.1 Endogenous test

While the occurrence of infectious diseases is typically regarded as an exogenous shock to households, it is imperative to investigate any potential bias that may arise from endogeneity. To address this concern, the study constructs an instrumental variable for infectious disease experience based on the historical incidence rates of legally notified infectious diseases at the provincial level from 2002 to 2009, and averaging these rates over the period. The incidence rates of legally reported infectious diseases in each province and the experience of infectious diseases at the village level are the occurrences of infectious diseases that are correlated, as they represent occurrences in distinct time periods. The incidence rates of infectious diseases in previous years mainly depend on natural conditions such as climate, hydrology, and geography, while the human environment in different regions also plays a role but is greatly influenced by historical changes, and the lagged variables are further selected to ensure the exogenous conditions. The incidence of legally reported infectious diseases is posited to have no direct impact on regional economic development, household income, or commodity prices, affecting only household consumption through the incidence of diseases, thus fulfilling the exogeneity requirement.

Table 3 presents the outcomes of instrumental variable estimation. The Durbin-Wu-Hausman (DWH) test showed a *p*-value of < 0.01, suggesting potential endogeneity in the experience of infectious diseases, as seen in column (1). The Kleibergen-Paap rk LM statistic of 98.39, along with the first-stage Kleibergen-Paap rk Wald *F*-statistic of 57.92, surpass the critical value at the 10% level of bias. Columns (1)–(3) exhibit the results of regression on household consumption 1, while Columns (4)–(6) illustrate the results of regression on household consumption 2. The instrumental variable findings affirm a significantly negative impact of infectious disease experience on household consumption at the 1% significance level, consistent with the baseline regression estimates.

**Table 3:** IV-2SLS regression result.

	**(1)**	**(2)**	**(3)**	**(4)**	**(5)**	**(6)**
	**consum1**			**consum2**		
Epidemic	−0.107^***^	−0.097^***^	−0.081^***^	−0.082^***^	−0.074^***^	−0.073^***^
	(−4.84)	(−3.74)	(−4.41)	(−5.43)	(−4.35)	(−5.12)
Controls	No	Yes	Yes	No	Yes	Yes
FE	No	No	Yes	No	No	Yes
Obs	1,539	1,539	1,539	1,539	1,539	1,539
*R*-squared	0.135	0.177	0.186	0.182	0.180	0.197
Kleibergen-Paap rk Wald *F*	57.92	59.29	57.50	61.23	60.01	69.33
Kleibergen-Paap rk LM	98.39	97.56	97.03	90.01	96.14	95.07
DWH Chi^2^	16.913	11.602	43.836	23.365	17.320	40.221
(*p*-value)	0.00	0.00	0.00	0.00	0.00	0.00

^***^, ^**^, ^*^ indicate significance levels of 1%, 5%, and 10%, respectively; *t*-values for standard errors clustered to the household level are in parentheses.

#### 3.2.2 Propensity score matching test

The Propensity Score Matching (PSM) test is employed to address the substantial difference in sample sizes between the treatment group (samples with infectious disease experience) and the control group (samples without infectious disease experience). This paper utilizes a 1:2 nearest neighbor matching and kernel matching, substituting household consumption 1 with household consumption 2 for analysis. The outcomes reported in Table 4 reveal that infectious disease experience induces a significant reduction in household consumption. This effect holds across different matching methods and changes in the dependent variable, reinforcing the robustness of the negative influence that infectious diseases exert on household consumption.

**Table 4:** Propensity score matching regression results.

**Method**	**Outcome Var**.	**Treat Gr**.	**Control Gr**.	**ATT**	**SE**	**t-value**
Neighbor matching	consum1	9.547	10.146	−0.599	0.020	−2.71^***^
	consum2	9.283	9.907	−0.624	0.014	−2.94^***^
Kernel matching	consum1	9.431	10.251	−0.820	0.056	−4.22^***^
	consum2	9.434	10.036	−0.602	0.059	−4.74^***^

Matching is only done for individuals within the common range of values; ^***^, ^**^, ^*^ indicate significance levels of 1%, 5%, and 10%, respectively; *t*-values for standard errors clustered to the household level are in parentheses.

#### 3.2.3 Other robustness tests

Initially, to address the potential skewing of regression results by extreme values, households with consumption levels in the lowest and highest 1 and 5% brackets are excluded from this study's analysis. The outcomes presented in columns (1) and (2) of Table 5 reveal that the negative effect of infectious disease experience on household consumption remains statistically significant at the 1% test level.

**Table 5:** Other robustness tests.

	**(1)**	**(2)**	**(3)**	**(4)**
	**Exclude 1%**	**Exclude 5%**	**Tobit model**	**Province FE**
Epidemic	−0.084^***^	−0.092^***^	−0.071^***^	−0.080^***^
	(−2.73)	(−2.80)	(−2.35)	(−2.85)
FE	Yes	Yes	Yes	Yes
Obs	1,523	1,462	1,539	1,539
*R*-squared	0.189	0.201	0.185	0.201

^***^, ^**^, ^*^ indicate significance levels of 1%, 5%, and 10%, respectively; *t*-values for standard errors clustered to the household level are in parentheses.

Additionally, concerning changes in the functional form, the paper applies the Tobit model with restricted dependent variables, employing maximum likelihood estimation due to the truncation of household consumption expenditures, which is greater than or equal to zero. The result, depicted in Column (3) of Table 5, indicates that the inhibitory effect of infectious disease experience on household consumption remains robust.

Finally, transitioning from household fixed effects to provincial fixed effects, the regression coefficients of the variable epidemic persistently exhibit statistical significance at the 1% significance level. This confirms the robustness of the benchmark regression results.

### 3.3 Long-term effect test

Research indicated that risk experiences can have lasting effects (28). This section uses four-period balanced panel data to test for the persistent impact of infectious diseases on household consumption. By the time of the 2014 survey, households had experienced a disaster in the short term. Subsequent surveys conducted in 2016, 2018, and 2020 allowed for an assessment of consumption behavior over extended periods, ranging from 3–5 years, 5–7 years, and 7–9 years post-disaster, respectively. Table 6 presents the consumption impact of infectious disease experience across distinct time intervals. The findings indicate that infectious disease experience significantly reduces household consumption levels for the 1–3 year, 3–5 year, and 5–7 year intervals. It is observed that the detrimental effect on consumption gradually attenuates with each successive year. However, this effect is no longer significant in the 7–9 years. A conceivable explanation for this phenomenon is the inherently abrupt and uncertain nature of infectious diseases, which can incite immediate panic and anxiety among individuals. This heightened emotional response leads to an excessive overreaction, characterized by an increase in savings and a concomitant decrease in immediate consumption. Over the longer term, the cognitive biases that drive such overreactions tend to attenuate. Consequently, household consumption exhibits increased resilience to disaster-induced shocks, with sensitivity to the impact of infectious diseases on total consumption expenditure diminishing over time. This pattern of resilience supports Hypothesis 2.

**Table 6:** Different time intervals effect test.

	**(1)**	**(2)**	**(3)**	**(4)**
	**1–3 years**	**3–5 years**	**5–7 years**	**7–9 years**
Epidemic	−0.061^***^	−0.029^**^	−0.013^**^	−0.011
	(−2.77)	(−2.23)	(−2.01)	(−0.84)
FE	Yes	Yes	Yes	Yes
Obs	6,156	6,156	6,156	6,156
*R*-squared	0.186	0.181	0.197	0.204

^***^, ^**^, ^*^ indicate significance levels of 1%, 5%, and 10%, respectively; *t*-values for standard errors clustered to the household level are in parentheses.

### 3.4 Heterogeneity test of healthcare resources

Access to healthcare resources is a significant determinant of household consumption, especially in the context of infectious diseases (21). Rural households often encounter varying levels of access to medical insurance and healthcare resources, which can influence their capacity to manage the impact of infectious disease outbreaks and, subsequently, their consumption behavior.

#### 3.4.1 Health insurance heterogeneity analysis

Engagement in health insurance programs serves as a critical instrument for governments to facilitate access to healthcare resources. By offering financial safeguarding, bolstering economic stability, and mitigating the motives for precautionary savings, health insurance plays a pivotal role in diminishing the adverse effects of infectious diseases on consumer behavior within households. To investigate the moderating effect of health insurance on infectious disease shocks, this paper introduces an interaction term between household participation in health insurance and the experience of infectious diseases. As seen in Column (1) of Table 7, the coefficient of infectious disease experience remains significantly negative. However, the coefficient on the interaction term between health insurance and infectious disease experience is significantly positive at the 5% test level. This finding validates that while households may not completely avoid the adverse impact of infectious disease shocks through health insurance participation, health insurance remarkably mitigates the decline in consumption levels for affected households. In essence, the protective function of health insurance effectively mitigates the negative effect of infectious diseases on household consumption.

**Table 7:** Heterogeneity test of healthcare resources.

	**(1)**	**(2)**	**(3)**
Epidemic	−0.480^***^	−0.178^**^	−0.198^***^
	(−2.98)	(−2.28)	(−2.69)
Medical	0.064^**^		
	(0.32)		
Epidemic × medical	0.399^**^		
	(2.05)		
Square		0.143^*^	
		(1.97)	
Epidemic × square		0.042^**^	
		(2.17)	
Doctor			0.102^*^
			(1.85)
Epidemic × doctor			0.093^***^
			(2.82)
Obs	1,539	1,539	1,539
*R*-squared	0.280	0.093	0.106

^***^, ^**^, ^*^ indicate significance levels of 1%, 5%, and 10%, respectively; *t*-values for standard errors clustered to the household level are in parentheses.

#### 3.4.2 Medical resource constraints heterogeneity analysis

Timely and effective access to medical resources is critical in lessening the severity and duration of infectious diseases. This, in turn, diminishes the economic impact, including income loss and medical expenses, often exacerbated by such diseases, thereby easing the financial strain on household consumption. In this study, the availability of local healthcare resources is quantified by examining the size of the largest medical facility (*square*) and the number of medical personnel (*doctors*) present in the village. The objective is to analyze how rural households respond to infectious diseases under varying healthcare resource supply conditions. As presented in Column (2) of Table 7, the coefficient of the interaction term between square and infectious diseases is significantly positive. This implies that a larger medical facility size in the village corresponds to a reduced impact of infectious disease shocks on rural households. The regression results based on the number of medical staff, shown in Column (3), align with those obtained from the analysis of medical facility size. These findings collectively suggest that the adverse consumption effects of infectious disease shocks are more pronounced in regions with more severe healthcare resource constraints (29). In other words, the effect of infectious diseases exacerbates inequality issues due to heterogeneity in households' access to medical resources.

### 3.5 Mechanism test

The theoretical analysis posits that the experience of infectious diseases may influence household consumption through four key mechanisms: the income constraint effect, medical expenditure crowding-out effect, risk perception effect, and precautionary savings effect. This section presents an empirical analysis of these proposed mechanisms. The results in columns (1)–(3) of Table 8 reveal several significant findings. Infectious diseases are associated with a notable decrease in household income, a rise in medical expenditures, and a reduction in spending on transportation, tourism, and recreational activities. These findings underscore that the experience of infectious diseases affects household consumption both indirectly, through reduced income and increased healthcare costs, and directly, by prompting social disengagement due to heightened risk aversion and anxiety, which in turn leads to constrained consumption choices. The evidence supports the hypothesis that the income constraint effect, medical expenditure crowding-out effect, and risk perception effect are channels through which infectious disease experiences influence household consumption. As seen in column (4), the coefficient of the interaction term between precautionary savings and infectious disease experience is significantly positive at the 1% test level. This suggests that as the motivation for precautionary savings decreases, the impact of infectious disease experiences on consumption gradually diminishes. It is also noteworthy that among all household types, rural agricultural households are disproportionately impacted by health shocks. This observation implies that households in rural areas with greater income volatility face more pronounced effects from infectious diseases, potentially intensifying inequalities in household consumption. In summary, the experience of infectious diseases suppresses household consumption through the income constraint effect, medical cost crowding-out effect, and risk perception effect, and is moderated by precautionary savings, thus confirming Hypothesis 3.

**Table 8:** Mechanism test.

	**(1)**	**(2)**	**(3)**	**(4)**
	**Income**	**Medical cost**	**Risk perception**	**Precautionary savings**
Epidemic	−0.099^***^	0.041^*^	−0.261^**^	−0.043
	(−4.25)	(1.95)	(−2.39)	(−1.55)
Epidemic × pre				0.089^***^
				(2.95)
Controls	Yes	Yes	Yes	Yes
Obs	6,159	6,159	6,159	6,159
*R*-squared	0.135	0.205	0.097	0.170

^***^, ^**^, ^*^ indicate significance levels of 1%, 5%, and 10%, respectively; *t*-values for standard errors clustered to the household level are in parentheses.

### 3.6 Differences in the impact of infectious diseases and other shocks

China is known for its vulnerability to a range of natural disasters, with droughts and floods being particularly impactful. This study, as presented in columns (1) to (3) of Table 9, evaluates the effects of infectious diseases, droughts, and floods on consumption. The findings reveal that experiences of both infectious diseases and droughts have a significant negative influence on individual consumption, while the impact of floods is not statistically significant. The impact of infectious diseases is more severe, likely due to the unpredictability and difficulty in managing outbreaks, which can swiftly degrade a household's economic situation and consequently lead to a more substantial decrease in consumption.

**Table 9:** Different shocks effect test.

	**(1)**	**(2)**	**(3)**	**(4)**	**(5)**
	**Infectious diseases**	**Droughts**	**Floods**	**Chronic diseases short-term**	**Chronic diseases long-term**
Coefficients	−0.072^***^	−0.052^***^	−0.006	−0.053	−0.081^***^
	(−5.45)	(−3.88)	(−0.24)	(−1.42)	(−3.12)
Controls	Yes	Yes	Yes	Yes	Yes
Obs	6,159	3,781	3,976	6,159	6,159
*R*-squared	0.260	0.290	0.320	0.385	0.240

^***^, ^**^, ^*^ indicate significance levels of 1%, 5%, and 10%, respectively; *t*-values for standard errors clustered to the household level are in parentheses.

When considering health-related shocks, the study also explores the effects of both infectious and chronic diseases on consumption behavior. Column (4) focuses on the short-term impact of chronic diseases on household consumption. The estimated coefficient for consumption is not statistically significant. Column (5) delves into the long-term impact of chronic diseases on household consumption and finds that they significantly lower household consumption over the long term. The persistent nature and extended duration of chronic diseases hinder sick households from recovering their labor capacity and income over time, resulting in a prolonged suppression of consumption demand. In conclusion, infectious disease shocks lead to a rapid decline in household consumption in the short term, whereas chronic diseases impact household consumption in the long term.

### 3.7 Household risk buffer effects analysis

Effective household risk management reduces household financial risks and ensures the financial stability of the household in the face of unforeseen events (30). The study introduces an interaction term between risk aversion instruments and experiences of infectious diseases for examining the smoothing effect of formal and informal risk aversion strategies. Firstly, the paper explores the role of liquid assets, which can be swiftly liquidated to alleviate liquidity constraints during times of uncertainty. The sample households are classified into HtM (hand-to-mouth) and nHtM (non-hand-to-mouth) consumers based on the adequacy of their liquid assets (31). HtM consumers are those whose liquid assets are less than or equal to half of their annual income. The result in column (1) of Table 10 reveals that the coefficient of the interaction term is negative at the 5% significance level, indicating that HtM consumers experience a more pronounced consumption deterrent after an infectious disease event compared to nHtM consumers. This finding suggests that households with substantial liquid assets have a greater ability to self-insure their consumption in the face of an infectious disease shock. Secondly, the study examines the role of pension insurance, which can enhance household disposable income through social transfers. Column (2) indicates that the interaction term between pension insurance and infectious disease experience is not significant at the 10% test level. This suggests that participation in pension insurance does not effectively mitigate the consumption disinhibition of households caused by infectious disease experiences. Finally, social capital is considered as a potential buffer for households with limited wealth and fewer risk protection mechanisms. Social capital is measured by the frequency of contact with non-cohabiting relatives (*social*) (25). Strong social capital households are defined as those with frequent interactions. Column (3) demonstrates that, while social capital significantly increases household consumption expenditures, it is not an effective means of moderating the impact of infectious diseases on household consumption. This may be attributed to the localized nature of disease outbreaks, which restricts the efficacy of social capital, typically characterized by mutual assistance among relatives and neighbors. Furthermore, the reliance on external resources by social capital and the presence of fundraising deadlines complicate the rapid mobilization of funds in response to a shock.

**Table 10:** Household risk buffer effects analysis.

	**(1)**	**(2)**	**(3)**
Epidemic	−0.045^***^	−0.162^***^	−0.144^**^
	(−3.18)	(−2.85)	(−2.27)
HtM	−0.103^***^		
	(−3.05)		
Epidemic × HtM	−0.355^**^		
	(−2.17)		
Pension		0.027^**^	
		(2.32)	
Epidemic × pension		−0.227	
		(−0.71)	
Social			0.236^***^
			(3.04)
Epidemic × social			0.024
			(0.15)
Obs	1,539	1,539	1,539
*R*-squared	0.118	0.340	0.123

^***^, ^**^, ^*^ indicate significance levels of 1%, 5%, and 10%, respectively; *t*-values for standard errors clustered to the household level are in parentheses.

## 4 Discussion

This paper employs the panel fixed effects (FE) regression model and instrumental variable (IV) method to investigate the impact of infectious disease experiences on rural household consumption based on data from the China Family Panel Studies (CFPS) datasets in 2014, 2016, 2018, and 2020. The findings revealed a gradual decline in the negative impact on consumption attributed to experiences with infectious diseases. Additionally, households with limited health insurance and greater constraints on healthcare resources faced more severe consumption reductions. The pathways through which infectious disease experiences influenced rural household consumption included income constraints, the crowding-out of medical expenses, and heightened risk perceptions, with the precautionary savings motive moderating these effects. The conclusions offer empirical support for policies aimed at unlocking rural consumption potential, strategically allocating medical resources across regions, and enhancing the risk resilience of rural households.

Firstly, the occurrence of infectious diseases is associated with a substantial reduction in household consumption levels, a finding that corroborates the results of Yao et al. (12), who demonstrated that uncertainty from such events leads to a pronounced shift toward saving and a diminished tendency for consumption. This outcome is further supported by previous studies (13–15), which highlighted the increased economic vulnerability of households during health crises and the potential for irregular consumption patterns or the risk of poverty traps. Moreover, it is interesting to note that the impact of infectious diseases is persistent, as evidenced by the existence of a significant negative relationship between the experience of infectious diseases and household consumption at 1–3, 3–5, and 5–7 years post-disaster, with this association weakening over time, becoming no more significant at around 7–9 years post-disaster. This is a similar observation to Kong et al. (28) in that the disaster experience has a persistent effect. The phenomenon can be attributed to the fact that sudden infectious disease outbreaks are often unforeseen, and this uncertainty prompts individuals to develop an exaggerated perception of risk, often responding to shocks with a greater magnitude of shock than the actual impact over a short period. However, in the long run, selective cognitive biases caused by overreaction diminish, rendering households less sensitive and more resilient to infectious disease shocks, leading to a recovery in consumption levels. Findings from this study will be able to assist policymakers for extending beyond immediate economic interventions and implementing robust, long-term social support mechanisms designed to strengthen the economic resilience and recovery of households affected by these events.

Secondly, the heterogeneity analysis reveals that medical insurance serves as a risk protection mechanism, thereby mitigating the impact of infectious diseases on household consumption. However, constraints on medical resources supply exacerbate the negative effect of infectious diseases on consumption. Health insurance is identified as an effective strategy for households to cope with health shocks (21), and this finding provides further empirical support on the effects of health insurance in the context of epidemics. Furthermore, regarding the heterogeneity among medical resource constraints, the adverse impact of infectious disease shocks on household consumption is more pronounced for those facing severe constraints on health resources or residing in remote and poor areas, corroborating previous conclusions (4). To mitigate the effects of infectious disease and address the resulting inequalities in medical resource utilization, it is imperative to augment investment in healthcare in rural regions, particularly in isolated and economically disadvantaged areas.

Thirdly, this study extends the understanding of the adverse effects of infectious diseases on consumption by elucidating the underlying mechanisms. The results of this study confirm that infectious diseases reduce household consumption levels mainly through income constraints, medical cost crowding-out, and risk perception channels, and are moderated by precautionary savings effects. These results align with those of previous research (9, 12) and contribute to a more nuanced theoretical framework for assessing the destructive potential of infectious disease outbreaks and informing scientific, targeted epidemic response strategies aimed at preserving public welfare and societal stability. Furthermore, the analysis highlights the issue of increased consumption inequality among rural households during epidemics. This insight underscores the necessity for policymakers to focus on addressing the interplay between poverty and disease among vulnerable populations, particularly in the context of disaster-induced shocks.

Fourthly, the analysis reveals a pronounced disparity in the effects of infectious diseases on household consumption compared to other types of shocks, such as natural disasters. The impact of infectious diseases is more severe, potentially due to the heightened uncertainty, wider reach, and prolonged duration associated with disease outbreaks, which are typically more difficult to anticipate and mitigate than natural disasters (6–8). Infectious diseases can rapidly degrade the economic conditions both within and outside the household, leading to more substantial declines in consumption. While infectious diseases prompt a rapid decrease in consumption, chronic illnesses tend to have a more serious impact in the long term. The study also evaluates the efficacy of various household risk mitigation strategies (30). It is found that liquid assets are instrumental in mitigating the negative effects of infectious disease experiences on household consumption. In contrast, pension insurance and social capital do not significantly contribute to cushioning the impact. This finding highlights the necessity of enhancing household wealth accumulation to strengthen their self-insurance capabilities against consumption shocks.

Drawing from these findings, the study proposes the following recommendations:

Initially, a comprehensive policy framework should be established, integrating short-term consumption stimulus measures with medium-to-long-term strategies aimed at enhancing the supply of consumer goods and services. This can be achieved by precisely targeting affected groups and employing a range of interventions, such as consumption vouchers, transfer payments, tax reductions, and credit investments, to expedite recovery and stabilize the short-term economy. Concurrently, governments should refine long-term mechanisms to foster consumption growth and reduce urban-rural disparities by reforming household registration, social security, taxation, and distribution systems. Enhancing consumption supply capabilities and improving the consumer environment in rural areas are also vital for unlocking the full consumption potential of these regions.

Subsequently, strategic allocation of medical resources is necessary to address regional healthcare disparities. The focus of medical insurance initiatives should evolve from merely expanding coverage to ensuring the quality of treatments. This includes a scientific distribution of resources between regions and increased investment in rural medical insurance, particularly in remote and impoverished areas. Integrating marginalized populations into the medical insurance network can be facilitated through measures such as upgrading medical facilities, enhancing medical equipment, recruiting additional healthcare professionals, and addressing regional inequities in insurance coverage. These efforts aim to reduce the unequal utilization of medical resources.

Finally, efforts should focus on diversifying income sources and promoting wealth accumulation to bolster rural families' financial stability and risk management capabilities. Enhancing liquid asset reserves and financial accessibility is essential for improving the ability to withstand economic shocks. Additionally, increasing education on risk prevention and coping strategies can mitigate panic and anxiety, stabilize family expectations, and reduce the incentive for precautionary savings, thereby empowering rural families to effectively manage health crises.

Although this study has made a detailed analysis of the relationship between experience of infectious disease and household consumption from a micro perspective, there are still some limitations. Initially, we chose a dummy variable of the presence or absence of infectious disease experience as the independent variable. Other indicators could be added for more meticulous research, such as the number of households that suffered from infectious diseases or the specific types of infectious diseases. In coming studies, the inclusion of these variables helps to analyze the intensive marginal effects of the impact of infectious diseases. Second, due to the limitations of the sample, this paper examined only the consumption responses of rural households. However, there are significant differences between urban and rural residents in terms of the living environment, economic status, consumption behaviors, and risk management tools that may lead to different analyses of the impact of infectious diseases. That needs to be dug up in follow-up studies. Finally, the heterogeneity analysis presented in this study was predominantly focused on healthcare resources. Future studies could explore the heterogeneity in individual responses to infectious diseases across different life cycles, income brackets, and levels of health literacy. Additionally, with the digital economy and online consumption continually evolving, examining the influence of infectious diseases on the transition between offline and online consumption patterns presents a significant area of research.

## Data availability statement

Publicly available datasets were analyzed in this study. The datasets for this study can be found in the China Family Panel Studies (CFPS) database (isss.pku.edu.cn/cfps/). The CFPS project was conducted by the China Social Science Survey Center of Peking University and publicly available.

## Author contributions

LH: Project administration, Writing – original draft, Investigation, Conceptualization. XX: Writing – original draft, Visualization, Software, Methodology. JY: Writing – review & editing, Supervision, Funding acquisition.

## Footnotes

^1^In the CFPS 2016, 2018, and 2020 surveys, the question was deleted.
